# Technology Prospecting on Enzymes: Application, Marketing and Engineering

**DOI:** 10.5936/csbj.201209017

**Published:** 2012-11-09

**Authors:** Shuang Li, Xiaofeng Yang, Shuai Yang, Muzi Zhu, Xiaoning Wang

**Affiliations:** a School of Bioscience and Bioengineering, South China University of Technology, Guangzhou 510006, China; b School of Life Science, General Hospital of PLA, Beijing 100853, China

**Keywords:** Industrial enzyme applications, enzyme consumption, enzyme production, Chinese enzyme market, enzyme engineering

## Abstract

Enzymes are protein molecules functioning as specialized catalysts for chemical reactions. They have contributed greatly to the traditional and modern chemical industry by improving existing processes. In this article, we first give a survey of representative industrial applications of enzymes, focusing on the technical applications, feed industry, food processing and cosmetic products. The recent important developments and applications of enzymes in industry are reviewed. Then large efforts are dedicated to the worldwide enzyme market from the demand and production perspectives. Special attention is laid on the Chinese enzyme market. Although enzyme applications are being developed in full swing, breakthroughs are needed to overcome their weaknesses in maintaining activities during the catalytic processes. Strategies of metagomic analysis, cell surface display technology and cell-free system might give valuable solutions in novel enzyme exploiting and enzyme engineering.

## 1. Introduction

Enzymes are natural catalysts. They are produced by living organisms to increase the rate of an immense and diverse set of chemical reactions required for life. They are involved in all processes essential for life such as DNA replication and transcription, protein synthesis, metabolism and signal transduction, *etc*. And their ability to perform very specific chemical transformations has made them increasingly useful in industrial processes.

Numerous reviews concerning different topics have been published, relating to strategies for enzyme engineering [1, 2], biocatalyst in organic synthesis [3], biofuels production [4], and selected aspects of bioprocesses [5]. Representative processes of DSM, BASF, and Lonza have been discussed with respect to technological and economical perspectives of industrial enzyme applications [6]. In the following, this review would focus on three points. First, attentions are given to the current status of representative enzyme applications in the field of the technical applications, feed industry, food processing and cosmetics, with the aim of understanding the enzyme impact on modern chemical industry. Second, efforts are made to draw a simple and clear scenario about the industrial structure of global enzyme market. General environment of demand and supply of Chinese enzyme market is critically analyzed. To assess on a realistic and sound basis, large amounts of information has been collected from various sources including books, periodicals, patent literatures, company's annual report, market research report and internet webpage. Although enzyme preparations have been used by mankind over a long history, breakthroughs are needed to extend their uses in broader areas with more superior performance. Recent advancements in novel enzyme engineering are briefly introduced in the last part, especially for metagenomics, surface display techniques and cell free systems.

## 2. Industrial Enzyme Applications

Enzymes are applied in various fields, including technical use, food manufacturing, animal nutrition, cosmetics, medication, and as tools for research and development. At present, almost 4000 enzymes are known, and of these, approximately 200 microbial original types are used commercially. However, only about 20 enzymes are produced on truly industrial scale. With the improved understanding of the enzyme production biochemistry, fermentation processes, and recovery methods, an increasing number of industrial enzymes can be foreseeable. The world enzyme demand is satisfied by about 12 major producers and 400 minor suppliers. Nearly 75% of the total enzymes are produced by three top enzyme companies, *i.e*. Denmark-based Novozymes, US-based DuPont (through the May 2011 acquisition of Denmark-based Danisco) and Switzerland-based Roche. The market is highly competitive, has small profit margins and is technologically intensive.

Table 1 gives the representative examples of enzyme applications based on different industrial sectors, and discusses the technical benefits in various fields. According to a research report from Austrian Federal Environment Agency [7], about 158 enzymes were used in food industry, 64 enzymes in technical application and 57 enzymes in feedstuff, of which 24 enzymes are used in three industrial sectors. Almost 75% of all industrial enzymes are hydrolytic enzymes. Carbohydrases, proteases and lipases dominate the enzyme market, accounting for more than 70% of all enzyme sales.

**Table 1:** Enzyme applications based on fields [1, 3, 8–11].

Application fields		Enzyme	Technical benefits
Technical Applications	Pulp and paper industry	Amylases	Cleaving starch molecules to reduce the viscosity for surface sizing in coatings, but not used for dry strength agent additive.
		Lipases	Deinking and to control pitch in pulping processes.
		Cellulases	Improving softness by hydrolyzing cellulose in fibers, creating weak spots in fibers, making fibers flexible.
		Mannanases	Degrading the residual glucomannan to increase brightness.
		Laccases	Bleaching to improve brightness.
		β-xylanases	Enhancing pulp-bleaching process efficiency.

	Textile industry	Amylases	Desizing efficiently without harmful effects on the fabric.
		Cellulases	Removing the fuzz and microfibers to give the fabric a smoother and glossier appearance.
			Loosening the indigo dye on the denim to give a slightly worn look.
		Pectinases	Destabilizing the outer cell layer to improve fiber extraction.
		Laccases, glucoseoxidases	Creating bleaching agent in whiteness.

	Laundry Detergents	Proteases	Hydrolyzing protein-based stains in fabrics into soluble amino acids.
		Lipases	Decomposing fatty material, such as fats, butter, sauces and the tough stains on collars and cuffs.
Amylases		Removing resistant starch residues.
Cellulases		Modifying the structure of cellulose fiber to increase the color brightness and soften the cotton.

Food processing	Dairy industry	Chymosin, lipases, lysozymes	Cheese manufacturing.
		β-galactosidases, lactases	Breaking down lactose to glucose and galactose in milk processing to avoid lactose intolerance.

	Baking industry	α-amylases	Degrading starch in flours and controlling the volume and crumb structure of bread.
		β-xylanases	Improving dough handling and dough stability.
		Oxidoreductases	Giving increased gluten strength.
		Lipases	Improving stability of the gas cells in dough.
		Proteases	Reducing the protein in flour.

	Juice industry	Amylases, glucoamylases	Breaking down starch into glucose.
		Clarifying cloudy juice, especially for apple juice.
		Pectinases	Degrading pectins which are structural polysaccharides present in the cell wall.
			Increasing the overall juice production.
		Cellulases, hemicellulases*	Acting on soluble pectin hydrolysis and on cell wall components with pectinases
			Lowering viscosity and maintenance of texture.
		Laccase	Increasing the susceptibility of browning during storage.
		Naringinase and limoninase	Acting on compounds that cause bitterness in citrus juices

	Starch processing	α-amylases	Cleaving α-1,4-glycosidic bonds in the inner region of the starch.
			Causing a rapid decrease in substrate molecular weight and viscosity.
		Pullulanases	Attacking α-1,6- linkages, liberating straight-chain oligosaccharides of glucose residues linked by α-1,4-bonds.
		Neopullulanases, amylopullulanases	Acting on both α-1,6- and α-1,4-linkages.
		β-amylases	Cleaving α-1,4-linkages from non-reducing ends of amylose, amylopectin and glycogen molecules.
			Producing low-molecular weight carbohydrates, such as maltose and “β-limit dextrin”.
		Glucoamylases	Attacking α-1,4-linkages and α-1,6-linkages from the non-reducing ends to release β-d-glucose
		Isoamylases	Hydrolyzing α-1,6-linkages in glycogen and amylopectin.
		Glucose isomerases	Catalyzing isomerization of glucose to fructose
		Glycosyltransferases	Transferring a segment of a 1,4-α-D-glucan chain to a primary hydroxy group in a similar glucan chain to create 1,6-linkages.
			Increasing the number of branched points to obtain modified starch with improved functional properties such as higher solubility, lower viscosity, and reduced retrogradation.

	Brewing industry	α-amylases	Hydrolyzing starch to reduce viscosity.
			Liquefying adjunct**.
			Increasing maltose and glucose content.
		β-glucanases	Hydrolyzing glucans into soluble oligomers and leading to lower viscosity and better filterability.
			Improving wort separation.
		Pullulanases	Hydrolyzing α-1,6 branch points of starch.
			Securing maximum fermentability of the wort.
		Amyloglucosidases	Increasing glucose content.
			Increasing 1% fermentable sugar in “light” beer.
		Proteases	Increasing soluble protein and free amino-nitrogen (FAN).
			Malt improvement.
			Improving yeast growth.
		Pentosanases, xylanases	Hydrolzing pentosans of malt, barley and wheat.
Improving extraction and beer filtration.		
α-acetolactate-decarboxylases (ALDC)		Converting α-acetolactate to acetoin directly.
		Decreasing fermentation time by avoiding formation of diacetyl.
		Making beer taste right.

Animal feeds industry		Xylanases	Degrading fiber in viscous diets.
		Phytases	Degrading phytic acid to release phosphorus, and liberating calcium, magnesium cations.
		Proteases (subtilisin)	Degrading protein into its constituent peptides and amino acids to overcome antinutritional factors.
		α-amylases	Digesting starch.

Organic synthesis industry		Hydrolases, such as lipases, nitrilases, nitrile hydratases, esterases, amidases	Acylation, deacylation, enantioseparation.
		Alcohol dehydrogenases, lactate dehydrogenases	Reduction of C-O and C-C bonds.
		Monooxygenases, formate alcohol dehydrogenases, dehydrogenases	Oxidation of alcohols and oxygenation of C-H and C-C bonds.
		Fructose 1,6-bisphosphate aldolases, Diels-Alderases	C-C coupling.
		α-fucosidases, sialidases	Glycosidic bonds.

Cosmetics industry		Oxidases, peroxidases, polyphenol oxidases	Hair dyeing.
		Protein disulfide isomerases, glutathione sulfhydryl oxidases, transglutaminases	Hair waving.
		Papain, bromelain, subtilisin	Giving gentle peeling effects in skin care.
		Amyloglucosidases, glucose oxidases	Toothpastes and mouthwashes.

* In accordance with the principles of the council of the European Union, hemicelulases and celulases are forbidden in Fruit Juice Directive (Council Directive 2001 / 112 / EC).

** Adjunct is starchy cereals such as maize, rice, wheat, sorghum, barley or pure starch materials added to the mash.

### 2.1 Enzymes in Technical Applications

Technical enzymes are typically used as bulk enzymes in detergents, textile, pulp and paper industries, organic synthesis and biofuels industry. Technical enzymes are valued at just over $1 billion in 2010 by several research associations. It is estimated that the technical enzymes market will increase at a 6.6% compound annual growth rate (CAGR) to reach $1.5 billion in 2015 with the highest sales in the leather market and bioethanol market [12].

Commercially available enzymes used in these areas are amylases, proteases, lipases, cellulases, xylanases and catalases, *etc*. Among these, α-amylases seem to be the most versatile enzymes in the industrial enzyme sector no doubt due to the abundance of starch, with the applications ranging from the conversion of starch to sugar syrups, and the production of cyclodextrins for the pharmaceutical industry. With increase in their application spectrum, the research is focused on developing new α-amylases with more thermophilic, thermotolerant and pH tolerant characteristics to improve starch gelatinization, decrease media viscosity, accelerate catalytic reactions and decrease the risks of bacterial contamination. The most thermostable α-amylase currently used in industrial processes is from *Bacillus licheniformis* [13]. It remains active for several hours at 90 °C. One extracellular enzyme from *Pyrococcus woesei* was isolated that is active between 40 °C and 130 °C with an optimum at 100 °C and pH 5.5 [14]. In spite of this, retaining high α-amylase activity at pH around 4.0 is still desired for industrial starch processing. But there seemed to be no great progress in essence and it needs some huge technological advances. Nevertheless, the structural and dynamic features of amylase may give some inspiration to understand or improve other enzymes’ thermostability, as the heat resistance is always a subject of unfailing interest [15].

In recent years the importance of lipases as industrial catalysts has grown steadily. It is well recognized that lipases have broad range of substrates, high regio- chemo- and enantioselectivity and relatively high stability in organic solvents [16]. These outstanding characteristics pave the way for their exploitation in organic synthesis: esterification, transesterification, aminolysis and oximolysis reactions. However, the use of lipases (and of most enzymes) in industrial processes is still limited by their low stability under operational conditions and low activity or specificity on particular or non-natural substrates. Two approaches are considered as the way to solve the problems: i) to search for novel enzymes or microorganisms from specific or extreme environments (extremophiles), *i.e*. extreme temperature, pH or salt concentration, or organisms that cannot be cultured in the laboratory. The metagenomics [17] has emerged as a powerful approach for discovering novel enzymes from non-characterized samples without any need to cultivate and/or isolate them. ii) to engineer already known enzymes by rational design or random mutagenesis. Several successful cases can be cited, such as the enhancement of the activity and enantioselectivity of *Candida Antarctica* A lipase toward a difficult substrate (ibuprofen ester) by combinatorial reshaping of the substrate binding pocket [18].

Cellulases are widely used in textile applications for many years, and again received additional consideration in the enzyme market owing to their powerful ability in the degradation of lignocellulosic feedstocks. The cost of cellulases is a significant technical barrier to the conversion of lignocellulosic biomass to fuels associated with commercializing processes[4]. Cellulase preparations’ cost reduction attributed to two main strategies [19, 20]: i) economical improvement in production of cellulase by process and strain enhancement, *i.e*. cheaper medium and alternative inducer system and ii) improvement in the specific cellulase performance or activity to reduce grams of enzyme for achieving equivalent hydrolysis by cocktails and component improvement. Many companies have devoted themselves to developing new cellulase preparations by using genetic techniques and have streamlined production of those enzymes. After two generations of Cellic^®^ release in 2009 and 2010, Novozymes launched a new enzyme for production of bioethanol from agricultural wastes and residues, called Cellic^®^ CTec3 in Feb 2012. The new enzyme product has been claimed to be 1.5 times better than the previous Novozymes’ Cellic^®^ CTec2 and five times less of enzyme dose compared to competing enzymes to make the same amount of ethanol [21]. Cellic^®^ CTec3 could make the cost of cellulosic biofuels to around $2.0/gal of ethanol, which is competitive with production of corn ethanol and gasoline.

### 2.2 Enzymes in Feed Industry

The use of enzymes in animal nutrition has an important role in current farming systems [22]. Feed enzymes can increase the digestibility of nutrients, leading to greater efficiency in feed utilization. Also they can degrade unacceptable components in feed, which are otherwise harmful or of little or no value. Another benefit is positive impact on the environment by allowing better use of natural resources and reducing on faecal nutrient level applied to land. For example, diets based on cereals such as barley, rye and wheat are higher in non-starch polysaccharides (NSPs), which can decrease the intestinal methane production when supplemented with NSP enzymes. Furthermore, proteases can substantially reduce the amount of non-protein nitrogen supplement in diets of animals, thereby reducing the excretion of urea into the environment.

Currently, feed enzymes available commercially by catalytic types are: 3-phytase, 6-phytase, subtilisin, α-galactosidase, glucanase, xylanase, α-amylase and polygalacturonase, and most for the swine and poultry segment [23]. Though research interest into the potential value of feed enzymes has occurred in the field of aqua-culture and ruminant nutrition, commercially viable versions have not been produced[24]. Additionally, development of heat stable, improved specific activity and some new NSP enzymes, and rapid, economical and reliable assays for measuring enzyme activity has always been the focus and been intensified recently [22].

The global market for feed enzymes is definitely one promising segment in the enzyme industry. It was estimated at around $344 million in 2007, and expected to reach $727 million in 2015 [25]. The use of enzymes as feed additives is restricted in most countries by local regulatory authorities [26]. Applications may therefore vary from country to country. Though some of the geographies have attained saturation, the market for feed enzymes globally in broad terms, is growing on, *e.g*. regions like U.S, China and South – East Asia still hold a high growth potential.

### 2.3 Enzymes in Food Processing

Today's consumers demand higher levels of quality in their foods in terms of natural flavor and taste not only in US and Europe, but also in the developing countries where consumption shifts away from staple sources of calories towards life enjoyment. This trend triggered the need for the development of enzymes applications in food processing. The food and beverage enzymes segment is expected to reach about $1.3 billion by 2015 with the highest sales occurred in the milk and dairy market [12]. A modest, expected decline of enzyme sales was seen in baking industry. While the concepts and need for the healthy foods promote the positive growth in the whole food enzyme market.

Food enzymes are mainly used in baking industry, fruit juice and cheese manufacturing, as well as wine making and brewing to improve their flavor, texture, digestibility, and nutritional value. In terms of enzyme regulations, enzymes used in food can be distinguished into food additives and processing aids. Most food enzymes are considered as processing aids only a few are used as additives, such as lysozyme and invertase [7]. From a regulatory point of view, distinction between processing aids and additives is very important because the national regulatory context of enzymes differs significantly in different countries, even among the EU Member States. But in general, the processing aids are used during the manufacturing process of foodstuff, and do not have a technological function demand in the final food. Enzymes used in food processing are typically sold as enzyme preparations, which contain not only the desired enzyme, but also metabolites from the production strain and several added substances such as stabilizers. All these materials are expected to be safe under the guidance of good manufacturing practice (cGMP).

The key item in evaluating enzyme preparations safety is the safety assessment of the production strain. Only about nine recombinant microorganisms are issued as Generally Recognized As Safe (GRAS) based on FDA regulations from a relatively small number of bacterial and fungal species primarily *A. oryzae*, *A. niger*, *B. subtilis* and *B. licheniformis*. In order to increase the enzyme production level, modifications including protease-deficient and sporulation-deficient were introduced the wild-type host microorganisms [27]. Olempska-Beer [28] reviewed the microbial strains engineered for food enzyme production from a security point of view.

### 2.4 Enzymes in Cosmetics

In a recent study carried out by market researcher Reportlinker, the market for enzymes used in cosmetic and toiletries is set to grow by 5% per year up to 2015 driven by the technological progress (achieving or maintaining enzyme stability and activity) and consumer awareness of seemingly potent power of enzymes. Although interests in the use of enzymes in cosmetics have been continuous increased, past and present areas of related applications seem to be few. One successful example is the use of superoxide dismutase (SOD) to capture free radicals and prevent damage to skin caused by environmental pollution, bacteria and other harmful factors. It is proposed to use a combination of SOD and peroxidase as free radical scavengers to reduce UV-induced erythema in sunscreen cream [29]. This application is highly expected partially attributed to this move towards organic compounds in place of petrochemical-based ingredients, which is highlighted by the increasing use of organic products [30]. Besides that, peroxidase is also used to prevent cosmetic formulations from bacterial attack for it can consume the oxygen present in the contents. Another example is proteases used in skin creams to clean and smoothen the skin by peeling off dead or damaged skin [31]. But there is a problem that it is difficult to stop the enzyme to eat the skin, which may cause skin irritations.

Patents search using “cosmetic” and “enzyme” as keywords reveals that more research focuses on the applications of enzymes in skincare after 1999. More than 150 patents were applied per year after 2003. These applications include catalase in skin protection [32], laccase in hair dye [33], lipase to prevent treat skin rash or diaper rash [34], endoglycosidase and papain in toothpaste and mouthwash to whiten teeth, remove plaque and odor-causing deposits on teeth and gum tissue [34], and vitamin precursor and fatty alcohol and some enzymes coupled with polymeric molecules [35]. In addition to all of the above, enzymes are also used in contact lens cleaners to remove protein films [36]. Larcabal [37] reported that although subtilisin A removed light deposits more effectively, papain was slightly better with medium deposits and more effective with heavy deposits than subtilisin A.

## 3. Global Enzyme Demand

**Figure 1:**
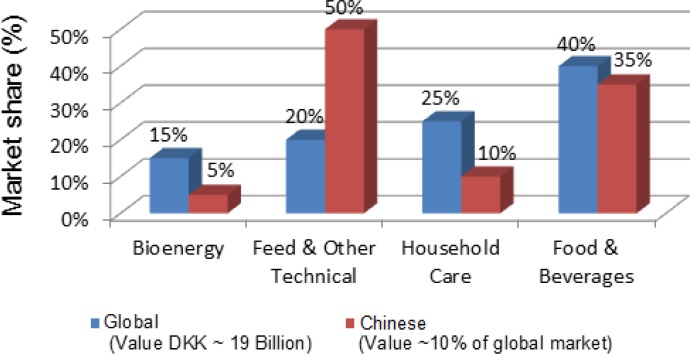
Chinese versus global enzyme market in 2010 (Roughly estimated) [39]

Global enzymes market is estimated to rise 7 percent at a healthy pace to $8.0 billion in 2015 [12]. Gains will reflect a continued world economy rebound from the global financial crisis of 2009. Enzymes are employed in a diverse array of applications in industries and scientific research, ranging from the degradation of various natural substances in the starch processing, detergent and textile industries, to the manipulation of DNA/RNA in biotechnology research. As illustrated in Fig. 1, the global enzyme market was dominated by the food and beverage industry, which benefits from the expansion of the middle class in rapidly developing economies. Growth came mostly from baking enzymes and other smaller applications such as fat and oil processing. However, the growth pace will be moderate as maturity in developed regions North America and Western Europe offsets faster growth in the Asia/Pacific and other developing regions. The fast growth over the past decade has also been seen in a wealth of other industries spanning from organic synthesis in pharmaceutical industry to diagnostics enzyme with expanded access to medical care in developing countries, and the advent of health care reform in the United States. Meantime the detergent industry, once the largest sector in the global enzyme market, experienced a decline due in part to the pricing pressures from the main detergent manufactures after the turn of the century. Demand for cleaning enzymes was accelerated by 2005 as the product lines were reformulated with more-effective new enzymes launched continuously. Bioenergy production enzyme demand was limited by the new legislative mandates for grain based ethanol. While the development of the second generation biofuels derived from cellulosic raw materials will be in favor of demand growth over a long time.

From a regional perspective, North America was, by far, the largest consumer of enzyme products followed by the second largest regional market of Western Europe. The developed regions adopted new enzyme technologies in industry applications at a torrid pace, as their companies invested in enzyme-related technologies to address environmental issues, improve productivity and increase product value. Nevertheless, North America and Western Europe will see the slower gains in enzyme consumption, restrained by the relatively mature markets. In particular, the American subprime lending crisis and the European debt crisiswill have a negative effect on enzyme consumption. However, the Asia/Pacific region will undergo a rapid increase in enzyme demand due to strength in China, Japan and India, reflecting the size and strength of these country's economics.

Sales of enzymes in China have grown at a fast pace in the past decades, accounting for roughly 10% of the global enzyme market in 2010 [39]. China was also the world's second largest enzyme market in 2010, although the America led demand by a wide margin. In China, the largest segment of enzyme market occurred in the feed and other technical applications accounting for about 50% (Fig. 1). It is different with the world enzyme demand patterns with the food and beverage processing occupying the largest market share. During 2008-2013, consumption of feed enzymes in China is expected to grow at an average annual rate of about 7.5%, increasing to 46.03 thousand metric tons in the year 2013 [25].

With the advancement of the animal production industries, demand for enzyme preparations increased rapidly. The Chinese government has been making plans for the use of phytase in recent years, about 20-40% of concentrated feed (excludes ruminant feed), to achieve better utilization of total plant phosphorus. Because large quantities of hay and straw are used as animal feeds in China, it is important to add cellulase and other lignocellulose-degrading enzymes to aid their digestion. Thus, the varieties of feed enzymes are also increased. The Ministry of Agriculture (MOA) of China has approved thirteen categories of feed enzymes so far (Error! Reference source not found.) [40]. The source microorganisms are mainly *Aspergillus niger, Aspergillus oryzae, Bacillus subtilis* and *Trichoderma longibrachiatum*, *etc*.

**Table 2:** MOA approved feed enzymes and their production sources [40].

No.	Enzymes	Microorganisms	Usage
1	Amylase	Aspergillus niger, Bacillus amyloliquefaciens, Bacillus licheniformis, Bacillus subtilis, Trichoderma longibrachiatum*, Aspergillus oryzae*	Corn silage, corn, corn gluten feed, soybean meal, wheat, wheat middlings, barley, grain sorghum, oat, pea, tapioca, millet, rice
2	Pullulanase	*Bacillus acidopullulyticus*	
3	α-Galactosidase	*Aspergillus niger*	Soybean meal
4	Cellulase	*Trichoderma longibrachiatum*	Corn, barley, wheat, wheat bran, rye, grain sorghum
5	β-Glucanase	*Aspergillus niger, Bacillus subtilis*, Trichoderma longibrachiatum, Penicillium funiculosum*	Wheat, barley, canola meal, wheat byproduct, oat groats, rye, triticale, grain sorghum
6	Glucose Oxidase	*Penicillium notatum*	Glucose
7	Lipase	*Aspergillus niger*	Plant and animal sources of fats and oils
8	Maltase	*Bacillus subtilis*	Maltose
9	Mannanase	*Bacillus lentus*	Corn, soybean meal, guar meal
10	Pectinase	*Aspergillus niger*	Corn, wheat
11	Phytase	*Aspergillus niger*, *Aspergillusoryzae*	Corn, soybean meal, sunflower meal, hominy, tapioca, plant byproducts
12	Protease	*Aspergillus niger, Aspergillus oryzae, Bacillus subtilisi*, Trichoderma longibrachiatum*	Plant and animal proteins
13	Xylanase	*Aspergillus oryzae, Humicolainsolens*, *Trichoderma longibrachiatum*, *Bacillus subtilis*, Penicillium funiculosum*	Corn, barley, rye, wheat, grain sorghum, triticale, oats

* Notes: Feed additive with the asterisk has obtained importation license. It cannot be manufactured in or exported to China until its safety, effectiveness and stability have been evaluated by Ministry of Agriculture.

The followed industrial users of enzymes in China are food & beverages and household care segments. The related enzyme market is growing due to macro trends, such as urbanization and GDP increase. It is estimated that the Chinese middle class will double in ten years. These city-dwellers have a strong position and are open for enzymes usage. The rising of personal disposable income makes Chinese consumers increasingly able to afford items ranging from advanced laundry detergent products to foods with higher-quality ingredients. For the development of enzymes used in household cleaning products, demand for cold active enzyme detergent formulations is high, so as to reduce the energy consumption and wear and tear of textile fibers. For example, Novozymes and Genencor, world leaders in the enzyme production, have launched about ten new products for laundry detergents to improve cleaning performance in lower temperature washes since 2005. While for the food industry, the growing unrest over food safety in China promoted people's demand for higher goods quality, safer production processing and improved nutritional value. Food is a local business and need to be present in the customers markets and create innovative solutions to meet the specific local preferences of customers. Thus, many international companies attached great importance to this field development. Novozymes Novamyl^®^ Steam is a new blend of enzymes that gives Chinese steamed bread increased and prolonged softness and freshness. In 2011, Royal DSM announced the joint with Yixing Qiancheng Bio-Engineering Co Ltd to strengthen food enzyme activities with solutions of guaranteed quality and consistency.

With food security concerns, China banned all new facilities using crops as a feedstock in 2007. The second-generation cellulosic biofuels from waste or inedible crops like jatropha are now regarded as the best option. Development of cellulosic ethanol fits well with the country's aim of generating 15% of its energy consumption from nonfossil fuel sources by 2020. In the “Biomass Development in the 12^th^ Five-Year Plan”, the Chinese government specially stressed on the investment and research of country's ethanol fuel, and speeding up the cellulosic ethanol industrialization. It plans to consume 5 million tons of ethanol between 2011 and 2015, which is nearly double that used during the previous five year period. In May 2010 three parties-Sinopec, COFCO and Novozymes - signed an agreement to jointly build cellulosic ethanol production project with capacity of 10,000 metric tons per year, in which Novozymes will supply with enzymes. The local ethanol producers are catching up. For example, Tianguan Group was building a 10,000-ton-per-year cellulosic ethanol plant using its own enzyme to convert cellulose to sugar.

## 4. Enzyme Production Structure

Commercial enzyme production has grown during the past decades in volume and number of products in response to expanding markets and increasing demand for novel biocatalysts, especially for the heavy environment burden. Global demand for enzymes is expected to rise almost 7% annually from 2010 to $8 billion in 2015 [12]. Gains over the historical period were attributed to the rapid increase in world energy prices, which made enzyme-related processes and products more cost effective, and facilitated the legislation of a rapid expansion of the fuel ethanol market, particularly in the US. Additionally, expanding middle classes in rapidly-growing developing countries contributed to robust gains in food and beverage enzymes, and the quick adoption of several enzyme-containing pharmaceuticals also supported growth. The recent introduction of Velaglucerasealfa (VPRIV™, approved on Feb 26, 2010) and Taliglucerasealfa (Elelyso™, approved on May 1, 2012), which are used for the treatment of rare genetic disorders, Gaucher's disease, has also led to significant growth in pharmaceuticals.

As international competition becomes increasingly sharp in enzymes market, strong companies aim to purchase other companies in order to become more efficient and competitive. In May 2011, DuPont acquired a majority stake in Danisco, including its Genencor division, which gives DuPont a strong position in the enzyme market, especially for the cellulosic ethanol production. Furthermore, Eli Lilly & Co.'s animal health division, Elanco, announced that it has acquired ChemGen early this year, to improve the development and commercialization of feed enzyme products used in poultry, egg, and meat production.

**Table 3:** Leading enzyme manufacturers.

Company	Location	Patent applications^a^	Established year	Major products	Market share^b^ (%)
Novozymes	Bagsvaerd, Denmark	902	1921	House hold care, food and beverage, bioenergy, Feed and other Technique enzymes, biophamaceuticals	47
Genencor	Copenhagen, Denmark	355	1982	Biofuels, food, animal nutrition, textiles, Detergents	21
DSM	Delft, the Netherlands	398	1952	Animal nutrition, food ingredients, personal care, pharmaceutical	6

Roche	Grenzacherstrabe, Switzerland	319	1896	Diagnostics, pharmaceuticals	20
Amano	Nagoya, Japan	147	1899	Pharmaceuticals, dietary supplement, biotransformation, diagnositics, food processing	
AB Enzymes	Feldbergstrasse, Germany	22	1907	Feed additives, food, textile, detergent, pulp & paper, biofuels	
BASF	Ludwigshafen, Germany	432	1865	Feed additives, pharmaceuticals, detergents		
Chr. Hansen	Horsholm, Denmark	12	1870s	enzymes for cheese	
Shin-Nihon	Aichi, Japan	2	-	Food, animal nutrition, biofuels	

ADM	Illinois, USA	2	1923	Food, feed, biofuels	5
KAO	Tokyo, Japan	409	1882	Beauty Care, human health care, fabric and home Care	
BioZyme	Joseph, MO	7	more than 50 years	Animal nutrition	

Verenium	San Diego, USA	75	-	Animal health and nutrition, grain processing, oilfield services	5
Iogen	Ontario, Canada	39	1970s	Biofuels, pulp & paper, textile, grain processing and brewing, animal feed	
Dyadic	Florida, USA	11	1979	Food, brewing & animal feed enzymes, biofuels, pulp & paper, textile Enzymes	
Meiji	Tokyo, Japan	136	1916	Food	
Enmex	Tlalnepantla, Mexco	1	1961	α-amylase, alkaline protease	
Nagase	Osaka, Japan	79	1832	Pharmaceuticals, food, agriculture, household, textiles	

a The data were from Derwent Innovations Index from 1981 to 2011 using enzyme as keyword.

b Some of the data are from the Danisco annual report for 2010 [41].

To date, the production of enzymes has been relatively concentrated a few developed nations located in Denmark, Switerland, Germany, Netherlands and USA (**Error! Reference source not found.**). Novozymes and Danisco in Denmark together serves an estimated 70% of the total enzyme market. For the new comer of DSM, the sales revenue accounted for 6% in 2010. Some Japanaese manufactures are playing an increasing important role in the world enzymes production. However, about one hundred companies produced enzymes in China with a total capacity of 700 thousand metric tons in 2010, which is estimated less than 1% of the world market share.There are still many small to medium-sized enzyme producers in China, which have been gradually washed out from the market. The enzyme industry is intent to develop this opportunity in China. Several multinational companies have invested in the enzyme industry in China. Novozymes (China) Co., Ltd. has three enzyme plants in China, one each in Tianjin, Shenyang and Taicang. Genencor Co., Ltd. (now part of DuPont) has an enzyme production base in China located in Wuxi. Recently, DSM announced to make a joint venture with a Chinese company, Yixing Qiancheng Bio-Engineering Company Ltd, to provide α-amylase and xylanase to acquire the food and beverage enzyme markets.

Enzyme industry in China has experienced over 50 years’ development since 1965 when China achieved industrialization of α-amylase in Wuxi. Generally speaking, China's enzyme products are not developed well in a sustainable way and cannot keep pace with superior product standards of many enzymes. These could be attributed to the aspects of product structure, applications, production scale, industry standard and R&D investment. For example, only about 20-30 types of enzymes are produced in China, which is much less product diversity than in developed countries. Even many domestic enzyme manufactures can only produce a single or a few products. In addition, the three major commercial enzymes – amylase, carbohydrase and alkaline protease, occupy more than 90% of the Chinese enzyme market, which does not meet the necessary requirement of product diversity. Besides unbalanced product types, limited application is another constraint. Although enzymes can be widely applied so many fields, enzymatic consumption in China is now quite confined to some certain application fields, mainly starch-processing (amylase and carbohydrase), detergent (alkaline proteases), beer and feed, which are late to bloom. Albeit with lower market shares, about 50 small Chinese enzyme producers occupied large part of the feed enzymes. And 4 largest Chinese producers jointly hold approximately 20% of the Chinese market [39].

**Figure 2:**
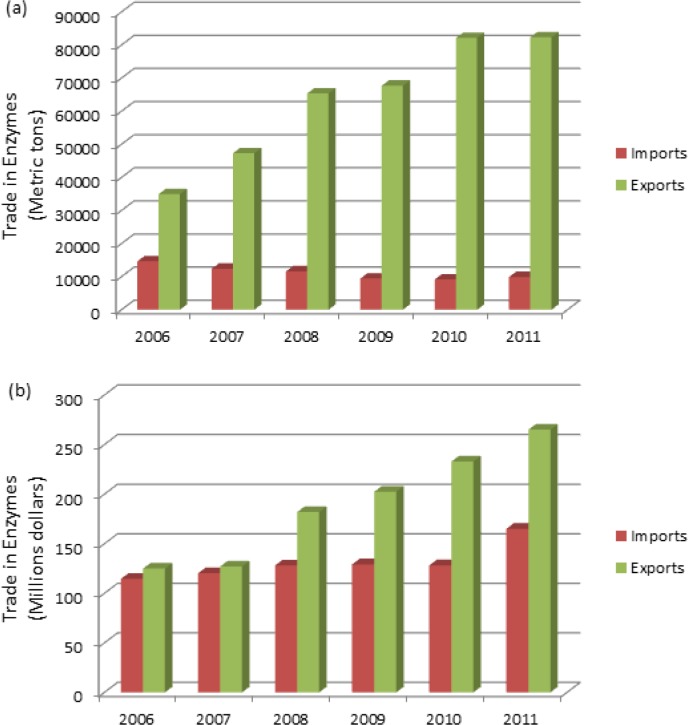
Import and export trends of enzyme in China (a) volumes in metric tons, (b) trade value in US dollars [42].

Today, China has become one of the most important enzyme suppliers and consumers around the world. The production and consumption capacity of enzymes in China are developed rapidly, which are increased more than two times from 2000 to 2010 (**Error! Reference source not found**.). After 2006, China transformed into a net exporter of enzymes by increasing the scale, quality and diversity of domestic production. The exported volume of enzymes continues to grow year by year (**Error! Reference source not found**.) [42]. By comparing the growth rate of export in volume (Fig. 2(a)) and value (Fig. 2(b)), it can be speculated that the major exported enzymes are most low value-added products. There is a big gap in the enzyme prices between imports and exports, which could not be narrowed in recent years. Recently, China has experienced very good market growth for thermostable amylase and glucoamylase. The major exported enzymes are high stable α-amylase, midrange thermal stable amylase, carbohydrase, phytase, and compound enzymes. The top global buyers are the United States, Japan, India and Korea. Going forward, China will continue to import large quantities of enzymes from abroad – especially high value-added products – but the pace of export growth will exceed that of imports, leading to an expanding trade surplus in part aided by investment by overseas-based firms in production facilities in China.

**Table 4:** Consumption and production of enzyme in China (thousands of metric tonnes) [43].

Year	Consumption	Production
1995	222.0	212.0
1996	230.0	230.0
1997	186.0	184.0
1998	223.3	221.0
1999	263.5	260.0
2000	274.7	272.0
2001	323.0	320.0
2002	351.8	350.0
2003	377.1	380.0
2004	412.0	420.0
2005	457.7	470.0
2006	479.7	500.0
2007	555.1	590.0
2008	561.2	615.0
2009	561.7	620.0
2010	577.0	655.0

Enjoying the strong growth momentum, Chinese enzyme industry also presented some other characters in the past, *e.g*. technology level kept being improved. The facts of local companies faced are in part a function of local producers’ inability to keep pace with advanced technologies need for their efficient production. The money invested in the R&D of most Chinese enzyme manufactures is not enough, accounting for 1-2% or less of that of annual sales. While for the global leader, Novozymes increased the investment in R&D by 8% in 2010 compared to the last year, representing 14% of sales, to support their long-term growth ambition [40]. Owing to inadequate R&D investment in technology innovation and new product development, none of Chinese domestic enzyme producers can rank among the top largest enzyme producers around the world, and this situation will remain in the near future. Only some advantages such as lower manufacturing cost and huge potential market may inject some confidence and development room in local producers. To overcome these obstacles for a sustainable enzyme industry development, domestic research institutes gave strong financial supports for the related research institutions in developing cutting-edge enzyme technology. China's policy makers have planned a large amount of budget on technology innovation and research on enzyme [44]. The 12^th^ five-year plan are focused on promotion of innovation and industrialization of biocatalysis, with particular emphasis on directed evolution, high expression, immobilization, coenzyme regeneration, multienzyme system, enzyme coupling with fermentation, asymmetric biosynthesis and nonaqueous biocatalysis, *etc*.

## 5. Advancements in Enzyme Engineering

Many worldwide corporations have recognized the bio-based technologies as one of the key drivers of sustainable growth. However, the biological process is often considered only when the chemical arsenal has failed to achieve synthesis of the target molecule. This is primarily because the unavailability of the desired enzyme to catalyze the reaction in an efficient manner. The exploitation of new types of enzymes, improvements of enzyme properties and of the production process are overall goals of innovation in the enzyme manufacturing industry. Accordingly, systematic methods in the field of enzyme and reaction engineering have allowed access to means to achieve the ends, *i.e*.1) screening for novel enzymes from natural samples with improved characteristics as a good starting point, 2) engineering the existing enzymes using genetic engineering approaches, 3) fining the enzyme processes in the enzyme manipulation to overcome catalyst limitation, *e.g*. downstream processing in enzyme manufacturing, formulationof enzyme preparations and enzyme immobilization, *etc*. Some recent developments and achievements in exploring new enzymes with outstanding properties will be briefly discussed in the following text.

### 5.1 Metagenomic analysis for novel biocatalysts recovery

In order to obtain microorganisms with special characteristics, traditional enrichment culture technique and screening of a wide variety of microorganisms for the desired activity are widely employed. However, Numerous microbes inhabit the biosphere, more than 99% of which are uncharacterized or unculturable due to difficulties in enriching and isolating microorganisms in pure culture [17]. Recent success of genome sequencing programmes has resulted in an explosion of information available from sequence databases, thus creating an opportunity to explore the possibility of finding new enzymes by database mining. Additionally, tools of metagenome analysis obviate the need of culturable microorganisms by directly extracting DNA from environmental samples and systematically screening for the open reading frames potentially encoding putative novel enzymes [17]. Metagenomic libraries screening are mostly based on the function-driven approaches: i) direct phenotypical detection [45], ii) heterologous complementation[46] and iii) induced gene expression [47]. By accessing different locations such as volcanic vents, deep oceans beds, arctic tundra, *etc*, this method could help people to find more than a million previously unknown genes coding for novel enzymes. Numerous studies by metagenome screening have yielded enzymes with potential for biocatalytic applications, such as nitrilase [48], lipase [49], β-lactamase [50], protease [45] and many others, which could serve as a good start for protein engineering [51]. However, the metagenome analysis method also suffers from disadvantages, such as missing constitutively expressed genes, sensitive to orientation of genes and not for substrates that do not migrate to the cytoplasm. The rapid development of cloning-independent DNA sequencing technologies and the combination of metagenomics, metatranscriptomics, and metaproteomics will promote the new biocatalysts discovery.

### 5.2 Surface display for high-throughput protein screening

Enzymes obtained by screening approaches often force the process engineers to alter process parameters due to inadequacies of the enzyme, such as instability, inhibition, narrow substrate specturm, low yield or selectivity. Utilization of enzyme improvement tools therefore provides a feasible means to fine tuning for adaptability to industrial scale production. Many different enzyme engineering and process engineering methods are available today, such as directed evolution, *de novo* protein design, use of nonconventional media, using new substrates for old enzymes, immobilization, active-site imprinting, *etc*. A large volume of literature on protein engineering and process engineering is availabe everywhere [2, 52–54]. Here, I would like to only focus on a tiny fraction of it.

Phage display is a powerful technique for screening large libraries of proteins which relates phenotypes with their corresponding genotypes. This method is particularly used in “synthetic binding protein engineering”, where libraries of synthetic binding proteins were developed with antigen-binding sites constructed from man-made diversity [55]. Jung et al [56] generated a random library of lipase displayed on the surface of *Escherichia coli* using the ice-nucleation protein (INP) as an anchor. The top mutant showed 29-fold increase in whole-cell activities compared to wild-type enzyme, and the lipase displaying cells can serve as an alternative immobilized biocatalyst in aqueous-organic solvent reaction medium. Yeasts are another attractive host strains for cell-surface display systems due to their safety, ease of high-cell density cultivation and the capability of eukaryotic proteins folding and glycosylation [57]. These properties make enzyme displayed yeast be of great value in biocatalytic process, especially in the production of biodiesel, polyester and esters using lipase displaying yeast as whole-cell biocatalysts [58–60]. In the review article, Wittrup [57] indicated that the approaches that yeast surface display coupling with fluorescence activated cell sorting (FACS) would be novel platforms for rapidly discovering new protein catalysts in industrial settings, due to without requiring soluble expression and purification.

### 5.3 Cell-free system applications in enzyme engineering

Cell-free expression systems were also described as important tools for protein engineering and production [61–63]. By removing the native genomic DNA and transport barriers (cell membrane/wall), processes occurring during the manipulation of cellular systems, like the cloning of a gene-construct into an expression vector system, cell transformation, cell culturing, cell lysis and the removal of cellular compartments are simply bypassed. The limitations of recombinant protein expression in living cells, such as protein degradation and aggregration will also be avoided [62]. Nakano [64] developed a novel strategy in an array format for generation and screening of protein library by affinity selection or enzymatic activity. Through ribosome display, another perspective cell-free technology for *in vitro* selection and evolution of proteins from large libraries, affinity of a human interleukin-13-neutralising antibody was improved by over 200-fold [65]. Additionly, *in vitro* compartmentalization (IVC) techniques in cell-free systems showed a strong momentum for the directed evolution of enzymatic activity. One striking example is an extremely fast phosphotriesterase was obtained by IVC lead to a 63-fold increase in *k*_cat_ [66]. A high-throughput IVC screening platform for oxygen-tolerant [FeFe] hydrogenases evolution was also developed and used to identify mutants. The mutant hydrogenase retains significantly higher methyl viologen reduction activity than the wildtype enzyme after oxygen exposure [67, 68].

## 6. Summary and Outlook

The beauty and charming of enzyme uses in industrial biotechnology are again and again exemplified. Aggregate demand for enzymes in the global market is projected to rise at a fast pace in recent years. Carbonhydrases will remain the leading enzymes in the next few years. While strong growth in the product category will be led by several markets, including food and beverage, animal feed and detergent industry. The pharmaceutical enzyme applications will see the fastest growth as growing per capita income in the developing regions lead to greater access to health care. America will remain the leading consumer of enzymes, while the Asia/Pacific region is forcased to surpass Western Europe as the second largest consumer of enzymes. The major contribution of the Asia/Pacific regions’ demand is attributed to China and India as the size and strength of these countries's economies growat a torrid pace. Western Europe would still occupy the largest producer of enzymes in the future ten years. However, Asia/Pacific companies, especially for the Japanaese and Chinese manufactures, are playing an increasing important role.

In the past decades, many sectors of chemical industry were restrained from embracing enzyme technology, largely because enzymes were considered as being too delicate to survive the extreme conditions in real reaction vessels. Actualization of enzyme applications in industrial processes requires high performance enzymes with specific characteristics, which will stimulate research to explore new avenues to overcome their weaknesses. Some of the stratagies in the field are exploiting novel enzymes from nature, improving existing catalytic properties, broadening specialized enzymes to serve new functions, optimizing formulation of enzyme preparations, or *de novo* designing biocatalysts. These approaches have provided valuable candidates for the biocatalytic processes. However, breakthroughs of enzyme products for biochemical technology should be recruited.
